# The Predictive Value of Aortic Calcification on Computed Tomography for Major Cardiovascular Events

**DOI:** 10.3390/jcm13144019

**Published:** 2024-07-10

**Authors:** David-Dimitris Chlorogiannis, Sumant Pargaonkar, Anastasios Apostolos, Nikolaos Vythoulkas-Biotis, Damianos G. Kokkinidis, Sanjana Nagraj

**Affiliations:** 1 Department of Radiology, Brigham and Women’s Hospital, Boston, MA 02115, USA; dchlorogiannis@bwh.harvard.edu; 2 Division of Hospital Medicine, Jacobi Medical Center, NYC H+H, Albert Einstein College of Medicine, New York, NY 10461, USA; 3 1st Department of Cardiology, School of Medicine, National and Kapodistrian University of Athens, Hippokrateion General Hospital of Athens, 11527 Athens, Greece; 4 3rd Department of Cardiology, School of Medicine, National and Kapodistrian University of Athens, Thoracic Diseases Hospital of Athens “Sotiria”, 11527 Athens, Greece; 5 Section of Cardiovascular Medicine, Yale University School of Medicine, New Haven, CT 06510, USA; 6 Division of Cardiology, Montefiore Medical Center, Albert Einstein College of Medicine, Bronx, NY 10467, USA

**Keywords:** aortic calcification, major cardiovascular events, TAVI, computed tomography, cardiovascular events

## Abstract

**Simple Summary:**

With the prevalence of cardiovascular disease continuing to grow, more novel markers for the identification of patients at risk are required. Extra coronary calcification in the ascending and/or descending thoracic aorta, aortic arch, and abdominal aorta has recently been identified as a method to quantify the extent of atherosclerotic cardiovascular disease, with its role in predicting major adverse cardiovascular events being unclear. In this review, we aim to summarize the existing literature regarding the predictive role of aortic calcification as calculated by computed tomography for the risk prediction of major adverse cardiovascular events.

**Abstract:**

As the prevalence of cardiovascular disease continues to increase, early identification of patients at high risk of major adverse cardiovascular events (MACE) using reliable diagnostic modalities is important. Transcatheter aortic valve implantation (TAVI) is a minimally invasive percutaneous procedure used to replace the aortic valve with a bioprosthetic one, often without the need for surgery. Extra coronary calcification in the ascending and/or descending thoracic aorta, aortic arch, and abdominal aorta has recently been identified as a method to quantify the extent of atherosclerotic cardiovascular disease. However, its definitive role in the prediction of MACE remains unclear. We performed a comprehensive review to summarize the current literature on the diagnostic and predictive value of thoracic and abdominal aortic calcification, as quantified in computed tomography, for the association, risk stratification, and prediction of MACE and after TAVI procedures. Despite increasing evidence, the predictive role of thoracic calcification still remains unproven, with a need for carefully tailored studies to confirm these findings.

## 1. Introduction

Cardiovascular disease (CVD) remains the leading cause of mortality and morbidity globally and carries the greatest disease burden. Despite considerable efforts to reduce its incidence [1], over the past three decades, the prevalence of CVD has nearly doubled, with an estimated prevalence of 523 million people [2]. Additionally, it is estimated that in the span of two years after diagnosis of coronary artery disease (CAD) or peripheral artery disease (PAD), one-in-ten patients will develop a major adverse cardiovascular event (MACE), including non-fatal stroke, non-fatal myocardial infarction, the need for revascularization, or cardiovascular death, leading to mean total healthcare costs that are three times higher than those without MACE [3]. Therefore, prompt identification, risk stratification, and management of patients with atherosclerotic cardiovascular disease (ASCVD) who are at high risk of MACE form the cornerstone of primary prevention.

The 2013 American College of Cardiology/American Heart Association Task Force on Practice Guidelines identifies coronary artery calcification (CAC), characterized by the deposition of calcium in the walls of coronary arteries in coronary computed tomography angiography, as the single-most useful factor for risk stratification for ASCVD in individuals at intermediate cardiovascular risk [4]. While the utility of CAC in ASCD event prediction is well-established across guidelines, large retrospective studies have reported an independent association between incidental extra-coronary calcification, particularly thoracic aorta calcification (TAC), and abdominal aorta calcification (AAC) on non-contrast computed tomography, and an increased risk of MACE [5,6]. Despite these findings, both the European Society of Cardiology (ESC) and the American College of Cardiology/American Heart Association (ACC/AHA) primary prevention guidelines do not include aortic calcification as a part of the stepwise approach in the individualized prevention strategies for MACE in ASCVD [7].

Taking the above into account, this review aims to evaluate the current clinical evidence on the use of thoracic and abdominal aortic calcification on computed tomography (CT) for risk stratification and prediction of major adverse cardiovascular events. 

## 2. Search Methodology

A comprehensive review of the literature was conducted using MEDLINE database for original studies evaluating the predictive value of thoracic or abdominal aorta calcification visualized on CT for MACE. The search algorithm is detailed in Supplementary Table S1, including full texts in English, published from inception until 1 June 2023.

## 3. Thoracic Aorta Calcification on Non-Contrast CT

**Figure 1:**
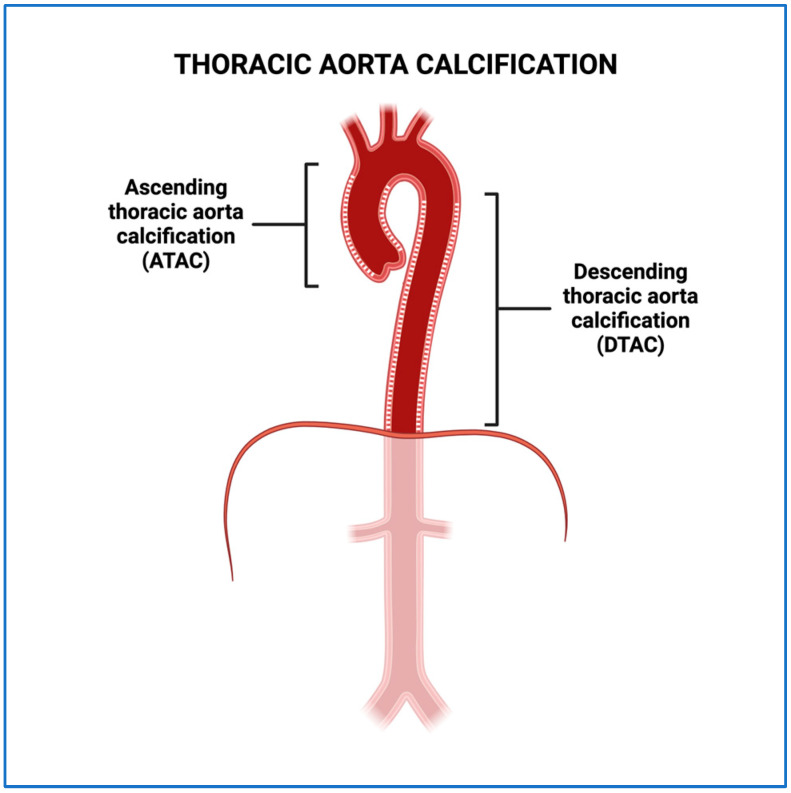
Ascending thoracic aorta calcification (ATAC) extending from above the level of the aortic annulus to the lower edge of the pulmonary artery. Descending thoracic aorta calcification (DTAC) from the lower edge of the pulmonary artery to the cardiac apex.

Thoracic aorta calcification is defined as the deposition of calcium in the intima and media layers of the ascending and/or descending thoracic aorta [8]. This can be stratified as ascending thoracic aorta calcification (ATAC; extending from above the level of the aortic annulus to the lower edge of the pulmonary artery) and descending thoracic aorta calcification (DTAC; from the lower edge of the pulmonary artery to the cardiac apex; Figure 1) [8]. Notably, much of the research evaluating the association between TAC and MACE stems from primary studies on coronary artery calcification [8]. 

### 3.1. Evaluation of TAC in the Multi-Ethnic Study of Atherosclerosis (MESA) Database

The MESA database is a prospective cohort study of the characteristics of subclinical cardiovascular disease (disease detected through non-invasive methods before clinical signs and symptoms become apparent) and the risk factors that predict progression to clinically overt cardiovascular disease. MESA includes 6814 individuals with ages ranging from 45 to 84 years old, from four ethnic groups (White, African-American, Hispanic, and Asia), with non-contrast computed tomography having been performed in all participants. While more than half of all patients in the MESA database had CAC, the prevalence of descending and ascending thoracic aorta calcification was 27% and 5%, respectively [8,9,10]. 

Although multiple studies evaluating the association between TAC and MACE have been conducted using the MESA database, the results are heterogenous. Thomas et al. studied 5887 patients from the MESA database to identify an association between ATAC volume (measured with ATAC Agatston score; high Agatston score means more calcium buildup) and MACE over a mean follow-up duration of 2.5 years. The investigators found that ATAC volume is associated with a significantly increased risk of coronary artery disease (hazard ratio (HR) 1.90, 95% CI 1.14–3.16, *p*-value < 0.05), ASCVD (HR 1.93, 95% CI 1.26–2.94, *p*-value < 0.05) and ischemic stroke events (HR 2.14, CI 1.21–3.78, *p*-value < 0.05) [8]. Another analysis by Budoff et al., which included 6807 patients from the MESA database, found an increased 5-year risk of coronary artery disease only in women with TAC (chi-square: 5.33, *p*-value < 0.05), irrespective of concurrent CAC presence [6].

Contrary to the above, several studies conducted on the MESA database have failed to demonstrate a significant association between TAC and MACE. In a study by Kim et al., in a median follow-up of 11.3 years, the authors evaluated 406 individuals with TAC vs. 3009 individuals, without TAC, and reported no significant difference for hazard ratios of coronary heart disease (CHD) and CVD (HR: 0.75, 95%CI: 0.35–1.60, *p*-value > 0.05) and HR: 1.17, 95%CI: 0.73–1.87, *p*-value > 0.05), respectively) between the two groups. This result was replicated for all-cause mortality, since individuals with TAC were not associated with increased risk for all-cause mortality (HR: 1.19, 95%CI: 0.83–1.70, *p*-value > 0.05) [11]. Yet, in a study by Kianoush et al., in 6805 MESA participants, using multivariate Cox regression models, the investigators found an independent association between TAC and ischemic stroke (Net classification improvement (NRI) 0.0023, *p*-value < 0.05) over a median follow-up duration of 12 years [12]. However, the predictive value of TAC for MACE appeared to be minimal and, therefore, insignificant beyond that provided by traditional risk factors (*p*-value > 0.05) [12]. These results were consistent with those of a previous study with 2303 asymptomatic adults, wherein the addition of TAC status to the Framingham risk score (FRS) or CAC status did not improve the prediction of MACE over a mean follow-up of 4.4 years (area under the curve (AUC) = 0.769, *p*-value > 0.05) [13]. Similarly, while evaluating a subgroup of 5745 non-diabetic patients with intermediate cardiovascular risk for concurrent CVD or CHD, the addition of CAC to the Framingham risk score prediction model improved discrimination (AUC_CAC_ = 0.712 vs. AUC_FRS_ = 0.643, *p*-value < 0.05) but not the addition of TAC. Notably, inclusion of TAC in the FRS  +  CAC model worsened the discrimination for incident CVD or CHD, hindering its ability for MACE prediction (AUC_TAC+CAC+FRS_ = 0.646, *p*-value < 0.05, respectively) [14].

### 3.2. Evaluation of TAC in Other Cohort-Based Studies for Stroke and Coronary Artery Disease Prediction

Beyond the MESA cohort, incidence of TAC and its association with MACE has been investigated in different cohorts around the world (Table 1). In 3930 subjects from the population-based Heinz Nixdorf Recall study, at a nearly nine-year follow-up, DTAC was independently associated with an increased risk of stroke (HR 1.11, 95% CI 1.02–1.20, *p*-value < 0.05), but not ATAC (HR: 1.02 95% CI 0.93–1.11, *p*-value > 0.05) [15]. These findings were similar to that of a sub-analysis of the Coronary Artery Calcium Consortium cohort of 41,066 patients, in which the presence of TAC was independently associated with increased mortality from stroke (HR 2.21 95% CI 1.39–3.49, *p*-value < 0.05) over a mean follow-up duration of 12 years [16].

In the community-based Framingham study, including 3486 individuals over a median follow-up duration of 8 years, Hoffman et al. found all extra coronary calcifications to improve prediction of MACE, independent of the Framingham risk score (HRTAC 1.40 95% CI 1.06–1.83, *p*-value < 0.05 and HRAAC 1.95, 95% CI 1.27–3.00, *p*-value < 0.05). However, when adjusted for CAC, the predictive value of extra coronary calcification for CAD lost statistical significance (HR 1.11 95% CI 0.83–1.47, *p*-value > 0.05) [17].

Also, stemming from the Heinz Nixdorf Recall study, conducted in a German patient population of 3630 subjects, Mahabadi et al. did not find the composite addition of TAC, epicardial adipose tissue volume, and left atrial index to the base regression model (adjusted with traditional risk factors and CAC) to improve the predictive accuracy for 10-year risk of MACE and death (improvement in c-statistic from 0.75 for the base regression model to 0.76). Notably, the improvement in c-statistic attributable to the addition of TAC was none, further highlighting its weak predictive value for MACE [18]. Yet, in another single-center study from the Netherlands, evaluating 567 patients with established CVD, the addition of TAC to different regression models did not improve the prediction rates for the four-year risk of MACE (NRI: (− 4.08, 95% CI− 9.97 to 3.95) [19]. Interestingly, in a small single-center cohort study of 390 consecutive patients referred for cardiac CT, the addition of the Agatston score of DTAC increased the 5-year predictive precision for CVD events of a multivariable Cox regression model, which included the Framingham risk score (HR: 1.06, 95% CI 1.02–1.09, *p*-value < 0.05) [20]. Similar results were also reported by another retrospective study of 1426 intermediate-risk patients, in which the addition of TAC volume and density improved the 4-year predictive model’s accuracy for MACE despite adjusting for traditional risk factors and CAC (AUC_before_ = 0.768 vs. AUC_after_ 0.814, *p*-value < 0.05) [21].

**Table 1:** Studies evaluating the association of thoracic aorta calcification and cardiovascular related outcomes.

Study	Year	Cohort	Participants (n)	TAC Assessment Score System	Cardiovascular Outcomes	Prognostic Value after Adjustment for Risk Factors	Aortic Calcium Site
Wong et al. [13]	2009	EISNER	2303	Agatston score	Myocardial infarction, cardiac death, late revascularization, stroke	No	TAC
Elias-Smale et al. [22]	2010	The Rotterdam Study	2521	Agatston score	Stroke	Yes	Aortic arch
Budoff et al. [6]	2011	MESA Study	6807	Agatston score	CHD	Yes, only in women	TAC
Elias-Smale et al. [23]	2011	The Rotterdam Study	2153	Agatston score	CHD, cerebrovascular events	Yes, only for coronary heart disease	Aortic arch
Bastos Gonçalves et al. [24]	2012	Meta-analysis	11,250	AAC-24, AAC-8	Cardiovascular events	Yes	AAC
Criqui et al. [25]	2014	MESA Study	1974	Agatston score	Cardiovascular morbidity and mortality	Yes	AAC
Yeboah et al. [14]	2014	MESA Study	5745	Agatston score	Coronary heart disease, cardiovascular disease, all-cause mortality	Yes, only for CHD and all-cause mortality	TAC
Hermann et al. [15]	2015	Heinz Nixdorf Recall Study	3930	Agatston score	Stroke	Yes, only for TAC and ATAC	TAC
Bos et al. [26]	2015	The Rotterdam Study	2408	Agatston score	All-cause mortality	Yes	Aortic arch
Hoffmann et al. [17]	2016	The Framingham Heart Study	3486	Agatston score	CHD, cardiac death, cerebrovascular events, all-cause mortality	Yes, only for all-cause mortality	TAC and AAC
Harbaoui et al. [27]	2016	Multi-center prospective Study	164	Hounsfield units of the delineated structures	Cardiac mortality, all-cause mortality, heart failure	Yes, only for cardiac mortality and all-cause mortality	TAC and AAC
Mahabadi et al. [18]	2016	Heinz Nixdorf Recall Study	3630	Agatston score	CHD, stroke cardiovascular death	No	TAC
Forbang et al. [28]	2016	MESA Study	997	Agatston score	CHD, cardiovascular disease, All-cause mortality	Yes, only for all-cause mortality	AAC
Forbang et al. [29]	2016	MESA Study	1413	Agatston score	Cardiovascular disease risk assessment	n/a	AAC
Kim et al. [11]	2017	MESA Study	406	Agatston score	CHD, cardiovascular disease, all-cause mortality	No	TAC
Kianoush et al. [12]	2017	MESA Study	6805	Agatston score	Cerebrovascular disease, stroke mortality	Yes, only for Ischemic stroke, total stroke, and TIA	TAC
Dudink et al. [20]	2018	Single-center Study	390	Agatston score	CHD	Yes, only for descending aorta	TAC
Thomas et al. [8]	2018	MESA Study	5887	Agatston score	CHD, atherosclerotic cardiovascular disease, ischemic stroke	Yes, only for CHD and ASCVD	ATAC
O’Connor et al. [30]	2019	Single-center Study	829	Agatston score	Myocardial infarction, stroke, cardiovascular death	Yes	AAC
Craiem et al. [21]	2020	Single-center Study	1426	Agatston score	CHD	Yes, inversely associated	TAC
Hamandi et al. [31]	2021	Single-center Study	431	n/a	Peripheral artery disease, CHD, cerebrovascular disease, all-cause mortality	Yes, only in high vs. low and high vs. moderate models	TAC
Obisesan et al. [16]	2021	The CAC Consortium	41,066	Agatston score	Stroke mortality	Yes, only for women	TAC
Van ’T Klooster et al. [19]	2021	The UCC-SMART cohort	567	Agatston score	Myocardial infarction, non-fatal stroke, vascular-related death	No	TAC

AAC: abdominal aortic calcification; ASCVD: atherosclerotic cardiovascular disease; ATAC: ascending aortic calcification; CAC: coronary artery calcification; CHD: coronary heart disease; EISNER: early identification of subclinical atherosclerosis using non-invasive imaging research; MESA: multi-ethnic study of atherosclerosis; TAC: thoracic aortic calcification; n/a: not available.

### 3.3. Thoracic Aorta Calcification and Transcatheter Aortic Valve Implantation (TAVI)

The predictive value of TAC examined not only for MACE prediction but also for the prognosis after transcatheter aortic valve implantation or replacement (TAVI/TAVR) [32,33]. Historically, for the treatment of severe aortic stenosis open-heart surgery was the sole treatment option [34]. However, in cases where extensive calcification of the thoracic aorta (porcelain aorta) exists, the surgical complications are increased, such as postoperative stroke [35,36,37,38,39]. Thus, less invasive techniques have come to the forefront, like TAVI, even for patients with severe aortic stenosis and calcification [40,41,42]. In a single-center retrospective cohort of 371 patients who underwent TAVI for severe aortic stenosis and were stratified according to TAC volume into low, moderate, and high categories, at 1 year, all-cause mortality was 6% in the low and moderate TAC categories and 16% in the high TAC group (*p*-value < 0.05). In addition, adjusted Cox regression analysis showed roughly three times higher risk of all-cause mortality in the high TAC vs. low TAC groups (HR 2.98, 95% CI 1.34–6.63, *p*-value < 0.05) [31]. Additionally, in a study by Harbaoui et al., in 164 patients who underwent TAVI, over a median follow-up duration of 565 days, ascending thoracic aorta, descending thoracic aorta, and abdominal aorta calcification statuses were incorporated into a multivariable Cox regression model and adjusted for major traditional risk factors for MACE. The authors concluded that extra coronary calcification was significantly associated with cardiac mortality (HR: 16.74; 95%CI 2.21–127.05 *p*-value < 0.05) and all-cause mortality (HR: 2.39, 95%CI 1.18–4.84; *p*-value < 0.05), but not with heart failure (HR: 1.84; 95% CI 0.87–3.90, *p*-value < 0.05), highlighting its significance in patient risk stratification and patient selection [27]. Lastly, overall atherosclerotic burden in the thoracic aorta has also been linked to post-procedural acute kidney injury. In this domain, in a cohort study of 210 patients who underwent TAVI, the prevalence of atherosclerotic burden was significantly higher in patients who developed acute kidney injury compared to those who didn’t. (Relative frequency: 87.5% (95% CI 75–90%) vs. 71.4% (95% CI 50–87.5%), *p*-value < 0.05) [43].

## 4. Aortic Arch and Abdominal Aorta Calcification 

Extra coronary calcification, which can also affect the aortic arch, is a well-established and is an independent risk factor for in-hospital cardiovascular mortality and stroke following surgical or endovascular repair of aortic pathologies [44,45,46]. In order to explore such associations, Elias-Smale et al. analyzed retrospective data of 2521 patients with aortic arch calcification from the Rotterdam Study. The Rotterdam Study is a population study originating from the Netherlands, which initially included 7983 patients aged 55 years and over, who are followed prospectively for identification of risk factors for numerous diseases, including cardiovascular diseases [22]. In the study by Elias-Smale et al., aortic arch calcification was associated with nearly three times higher odds for cerebrovascular events compared to patients without aortic arch calcification, after adjusting for traditional ASCVD risk factors (OR 3.3 95%CI 1.5–7.4, *p*-value < 0.05). However, when adjusted for calcification in different vascular territories, the association lost its significance [22]. Lastly, a sub-analysis of 2408 elderly patients from the same cohort found aortic arch calcification to be strongly associated with increased cardiovascular-related mortality, independent of calcification in other territories (HR 1.75, 95% CI, 1.13–2.72, *p*-value < 0.05) after 15,775 person-years [26].

In an effort to accurately predict 5-year CVD risk, Elias-Smale et al., added status of aortic arch calcification to the Framingham risk score model in 2153 asymptomatic individuals from the Rotterdam Study. Over a mean follow-up duration of 3.5 years, the refitted model’s C-statistic improved from 0.69 to 0.74 for CHD risk, without improvement in stroke risk (HR 1.5 95% CI 0.8–3.1, *p*-value < 0.05) [23].

By examining 1413 participants from the MESA cohort, Forbang et al. found that abdominal aorta calcification volume was associated with traditional CVD risk factors, such as increasing age, smoking, increased body mass index, and reduced HDL cholesterol [29]. In addition, post-stratification with CAC underlined that subclinical abdominal aorta calcification showed an equal predictive value to CAC for hard coronary heart disease and atherosclerotic cardiovascular disease events (CHD events: AAC HR: 2.38, CAC HR: 4.35, *p*-value < 0.05, and ASCVD events: AAC HR: 2.66, CAC HR: 2.90, *p*-value < 0.05). Moreover, the authors also found a stronger association between AAC and CVD mortality compared to that between CAC and CVD mortality (AAC HR: 5.89, *p*-value < 0.05, CAC HR: 2.10, *p*-value < 0.05). Lastly, the authors concluded that although the predictive values of AAC and CAC were independent, the overall risk prediction was more accurate when both parameters were incorporated (AUC_hardCHD_ 0.805, *p*-value < 0.05, AUC_hardASCVHD_ 0.796, *p*-value < 0.05, and AUC_CVDMortality_ 0.821, *p*-value 0.053) [25]. In this context, by using retrospective data of 997 patients from the MESA cohort, Forbang et al. analyzed the calcium density quantified with the Agatston score in adjusted models with other CVD factors. The authors highlighted that AAC density failed to exhibit adequate 9-year predictive power for cardiovascular events (HR 1.07, 95%CI 0.36, 3.14, *p*-value < 0.05) [28]. This finding is in contrast to the results of a meta-analysis that underlined a stepwise increase in the relative risk in patients with higher AAC burden for all the study’s endpoints (coronary events relative risk (RR) 1.81, 95% CI 1.54–2.14, *p*-value < 0.05, cerebrovascular events RR 1.37, 95%CI 1.22–3.54, *p*-value < 0.05, and cardiovascular mortality RR 1.72, 95%CI 1.03–2.86, *p*-value < 0.05) in at least 2-year follow-up. However, the generalizability of the study was hindered by the inclusion of studies with high heterogeneity (I2 > 75%) [24]. Lastly, in a retrospective study of 829 patients over a mean follow-up duration of 11.2 years, CT-based AAC was a strong predictor of future cardiovascular events when used as the sole predictor (AUC: 0.67, 95%CI, 0.60–0.75, *p*-value < 0.05). The result was replicated even when combined with the Framingham risk score (AUC_univariate_: 0.76, 95%CI 0.72–0.81, *p*-value < 0.05, AUC_combined_: 0.78, 95%CI 0.73–0.82, *p*-value < 0.05) [30].

## 5. Conclusions

Despite accumulating evidence favoring an association between the volume of extra coronary calcification and the risk of MACE, significant heterogeneity between studies and prediction models precludes the generalizability of findings. This also prevents the applicability of findings to routine clinical practice. Large umbrella review studies might be useful to summarize the findings in order to conclude about the best possible use of extracoronary CAC for MACE risk prediction.

## Supplementary Materials

The following supporting information can be downloaded at: https://www.mdpi.com/article/10.3390/jcm13144019/s1, Table S1: Search Treatment Algorithm to detect studies that merit reference.
