# Supplementary material for: The Predictive Value of Aortic Calcification on Computed Tomography for Major Cardiovascular Events
Source: J Clin Med. 2024 Jul 10;13(14):4019. doi: 10.3390/jcm13144019 (PMC11277087; doi:10.3390/jcm13144019)
Supplement: Supplementary file 1 [file jcm-13-04019-s001.zip › jcm-3061925-supplementary.pdf]

Supplementary Table S1: Search Treatment Algorithm to detect studies that merit reference.

Database Screened and Results	Search Terms
**MEDLINE** (1,522 Results)	*("aorta"[MeSH Terms] OR "aorta"[All Fields] OR "aortas"[All Fields] OR "aortas"[All Fields] OR "aortae"[All Fields] OR ("aorta, thoracic"[MeSH Terms] OR ("aorta"[All Fields] AND "thoracic"[All Fields]) OR "thoracic aorta"[All Fields] OR ("aortic"[All Fields] AND "arch"[All Fields]) OR "aortic arch"[All Fields]) OR "aorta"[MeSH Terms]) AND ("calcific"[All Fields] OR "calcificated"[All Fields] OR "calcification, physiologic"[MeSH Terms] OR ("calcification"[All Fields] AND "physiologic"[All Fields]) OR "physiologic calcification"[All Fields] OR "calcifications"[All Fields] OR "calcinosis"[MeSH Terms] OR "calcinosis"[All Fields] OR "calcification"[All Fields]) AND ("j comput tomogr"[Journal] OR "commun theory"[Journal] OR "child teenagers"[Journal] OR "cancer ther"[Journal] OR "ct"[All Fields] OR ("tomography, x ray computed"[MeSH Terms] OR ("tomography"[All Fields] AND "x ray"[All Fields] AND "computed"[All Fields]) OR "x-ray computed tomography"[All Fields] OR ("computed"[All Fields] AND "tomography"[All Fields]) OR "computed tomography"[All Fields]))*
